# Rivaroxaban-Related Traumatic Large Subcutaneous Hematoma in the Calf Requiring Surgical Repair in an Elderly Patient

**DOI:** 10.1055/s-0040-1710359

**Published:** 2020-05-10

**Authors:** Yuki Tsujimoto, Goshi Matsuki, Yuki Noboru, Yoichi Nishii, Shinsaku Imashuku

**Affiliations:** 1 Division of Plastic Surgery, Uji-Tokushukai Medical Center, Uji, Kyoto, Japan; 2 Department of Surgery, Uji-Tokushukai Medical Center, Uji, Kyoto, Japan; 3 Department of Laboratory Medicine, Uji-Tokushukai Medical Center, Uji, Kyoto, Japan

The direct oral anticoagulant (DOAC) rivaroxaban (RIV) is a factor Xa inhibitor which is mainly used for the prevention of atrial fibrillation-induced ischemic stroke. 1 Recently, RIV has also been used in cases of aneurysm-associated chronic disseminated intravascular coagulation (DIC), although this application is off-label. 2 The use of DOACs is associated with uncontrollable hemorrhagic complications and even death. 3 4 5 Regarding the site of RIV-related bleeding complications, the J-ROCKET AF study in Japan reported bleeding in the upper gastrointestinal tract tract (6%), intracranial (5%), intraarticular (4%), and intraocular (3%) regions. 6 Therefore, at the time of DOAC-related major bleeding complications, rapid and reliable reversal of the anticoagulant effect is required, particularly in patients with life-threatening intracranial hemorrhage. 3 4 In such cases, 4-factor prothrombin complex concentrate (4F-PCC) has been employed, 4 5 7 though andexanet alfa was recently developed for reversal of factor Xa inhibitors. 8 Here, we report a case of traumatic severe subcutaneous bleeding in the calf of an elderly patient treated with RIV for chronic DIC due to thoracoabdominal aneurysms.

**Fig. 1:**
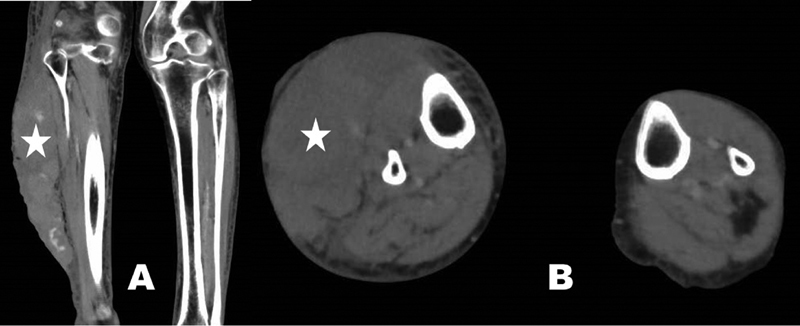
Contrast-enhanced computed tomography shows a large hematoma (indicated by the star) in the right calf 6 hours after trauma; ( **A** ) coronal view, ( **B** ) axial view.

**Fig. 2:**
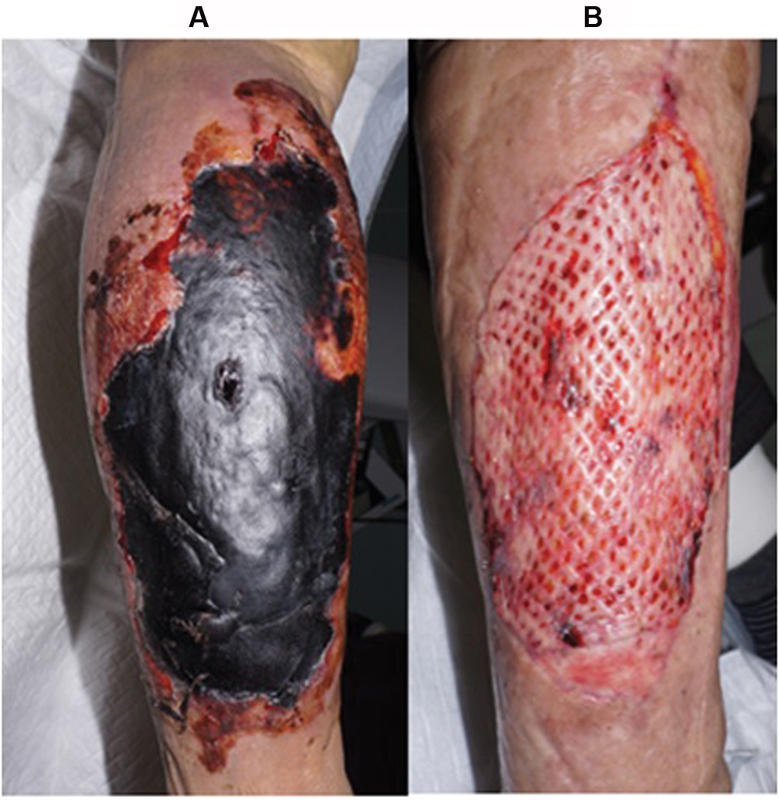
( **A** ) Necrotized and blackened skin of the right calf on Day 13 prior to debridement and artificial skin grafting; ( **B** ) successful skin grafting on Day 50.

An 84-year-old female who had undergone thoracic endovascular aortic repair for an aortic dissected aneurysm was suffering from chronic DIC. After being treated with intravenous and subcutaneous heparin, the patient became intolerant of subcutaneous heparin administration because of pain and was transitioned to oral RIV prior to discharge from hospital. Outpatient clinic assessment showed that her DIC was well controlled by the treatment with RIV (15 mg/d) and tranexamic acid (TXA; 1,500 mg/d). One year later, she suffered an accidental blunt injury to her right calf at home caused by a plastic costume case. She was hospitalized 3 hours later, at which point her hemoglobin (Hb) level was 9.0 g/dL, platelet count of 90 K/μL, serum blood urea nitrogen 24.4 mg/dL (reference; 7.8–18.9), and creatinine 0.89 mg/dL (reference; 0.45–0.82); prothrombin time (PT) was 29.7% (reference: 80–100%), PT-international normalized ratio 2.07 (reference: 0.9–1.1), and activated partial thromboplastin time 40.5 seconds (control: 27.9 seconds). Six hours after the injury, contrast-enhanced computed tomography (CT) scanning showed a large subcutaneous hematoma in her right calf causing swelling to 1.8-fold the width of the left calf ( Fig. 1 ). No arterial damage was detected and palpation of the dorsal artery of the right foot was possible. The bleeding was not considered to be life-threatening and the patient was kept under observation. On the following morning (Day 2 of hospitalization), RIV was discontinued but TXA treatment was maintained. Her Hb level dropped to 6.3 g/dL and a marked exacerbation of the hematoma size was observed. Although the patient received packed red blood cells (PRBC; four units) and fresh frozen plasma (FFP; four units), the skin over the hematoma developed large bullae, which became necrotized probably due to disruption of the perforating branch artery that provides nutrients to the cutaneous tissue. On Day 3, the Hb level remained at 7.3 g/dL, necessitating the administration of additional PRBC (two units) and FFP (two units). On Day 4, since the subcutaneous hematoma was thought to be further increased in size, treatment with PRBC (four units) and FFP (four units) was given combined with 4F-PCC (30 IU/kg, total: 1,000 IU). On Day 5, the patient's Hb level was stable at 9.3 g/dL and complete hemostasis was assumed to be achieved. The patient subsequently required plastic surgery to repair the necrotized and blackened skin on the right calf ( Fig. 2A ). On Day 13, debridement of the tissue was performed and artificial dermis was used to cover the wound followed by permanent skin grafting on Day 35. During this period, RIV was discontinued for 40 days; DIC was effectively managed with TXA alone and no thrombotic events occurred. The patient was able to walk and was discharged on Day 42. On day 50, successful skin grafting was confirmed ( Fig. 2B ).

DOAC-related major or severe bleeding is defined as hemodynamic instability, a fall in Hb level of 2 g/dL, or hemorrhage requiring blood transfusion. 7 The case reported here fits these criteria. The in vivo half-life of RIV is approximately 5 to 13 hours, and on the day of the injury, the patient had taken RIV (15 mg) and TXA (500 mg) in the morning before suffering the calf trauma. Six hours after the accident, CT scanning showed that the right calf had swollen to approximately twice its normal width due to the presence of a severe subcutaneous hematoma. RIV was withdrawn the next day but the bleeding continued and did not seem to be responding to the management with PRBC and FFP alone. In reversing DOACs, limited effectiveness of FFP was previously reported 9 while effectiveness of PCC was established. 10 In our case, initially we were hesitant to give PCC considering its thrombotic risk. Theoretically, complete metabolism of RIV can be achieved in less than 3 days from its half-life (5–13 hours) in an individual with normal renal function. Though PCC was thought to be clinically effective because of no more Hb drop, administration of it on Day 4 was late as an anti-RIV. Thus, bleeding cessation might have occurred due to improved clotting in the setting of chronic DIC. Anyway, eventually the case reported here required repair of the damaged skin. Optimal timing of the PCC administration is critical but remains controversial 5 7 11 due to the risk of thrombotic events, which may develop within 14 days of PCC use, 7 though in this case, no thrombotic events occurred. In summary, to prevent RIV-related severe bleeding complications and to limit the adverse consequences, timely administration of PCC or a new agent such as andexanet alfa 8 without relying on FFP is important to establish hemostasis.
